# Using a Dynamic Causal Model to validate previous predictions and offer a 12-month forecast of the long-term effects of the COVID-19 epidemic in the UK

**DOI:** 10.3389/fpubh.2022.1108886

**Published:** 2023-01-06

**Authors:** Cam Bowie, Karl Friston

**Affiliations:** ^1^Retired, Axminster, United Kingdom; ^2^Wellcome Centre for Human Neuroimaging, University College London, London, United Kingdom

**Keywords:** Dynamic Causal Model, COVID-19 mitigation measures, acute-post-COVID-19, hospital admissions, mortality incidence

## Abstract

**Background:**

Predicting the future UK COVID-19 epidemic provides a baseline of a vaccine-only mitigation policy from which to judge the effects of additional public health interventions. A previous 12-month prediction of the size of the epidemic to October 2022 underestimated its sequelae by a fifth. This analysis seeks to explain the reasons for the underestimation before offering new long-term predictions.

**Methods:**

A Dynamic Causal Model was used to identify changes in COVID-19 transmissibility and the public's behavioral response in the 12-months to October 2022. The model was then used to predict the future trends in infections, long-COVID, hospital admissions and deaths over 12-months to October 2023.

**Findings:**

The model estimated that the secondary attack rate increased from 0.4 to 0.5, the latent period shortened from 2.7 to 2.6 and the incubation period shortened from 2.0 to 1.95 days between October 2021 and October 2022. During this time the model also estimated that antibody immunity waned from 177 to 160 days and T-cell immunity from 205 to 180 days. This increase in transmissibility was associated with a reduction in pathogenicity with the proportion of infections developing acute respiratory distress syndrome falling for 6–2% in the same twelve-month period. Despite the wave of infections, the public response was to increase the tendency to expose themselves to a high-risk environment (e.g., leaving home) each day from 33–58% in the same period.

The predictions for October 2023 indicate a wave of infections three times larger this coming year than last year with significant health and economic consequences such as 120,000 additional COVID-19 related deaths, 800,000 additional hospital admissions and 3.5 million people suffering acute-post-COVID-19 syndrome lasting more than 12 weeks.

**Interpretation:**

The increase in transmissibility together with the public's response provide plausible explanations for why the model underestimated the 12-month predictions to October 2022. The 2023 projection could well-underestimate the predicted substantial next wave of COVID-19 infection. Vaccination alone will not control the epidemic. The UK COVID-19 epidemic is not over. The results call for investment in precautionary public health interventions.

## Research in context

### Evidence before this study

PubMed and Google searches failed to find any publication analyzing the COVID-19 pandemic using a Dynamic Causal Model except the one developed at UCL. PubMed and Google searches failed to find projections of 12 months or more of the epidemic in the UK. The three research units (IHME, ICL, and Youyang Gu) which publish regular updates of the COVID-19 epidemic besides the DCM project from UCL project forward a maximum of 120 days.

### Added value of this study

A three-month projection is insufficient to assess the full burden of anticipated disease due to the epidemic for which a full year projection is needed. This study does this and assesses the validity of a previous 12-month projection carried out a year ago. The research illustrates the methods and a sample of the many outputs generated by the model.

### Implications of all the available evidence

DCM offers a new way of trying to understand an epidemic caused by an infection such as the changes in transmissibility. The results indicate that the virus is going to persist and cause much morbidity and mortality over the next 12 months.

## Background

In October 2022, the predictions carried out 12 months earlier using a Dynamic Causal Model were assessed and found to underestimate the waves of new COVID-19 infections by 43%, deaths by 20%, tests by 24%, hospital admissions by 31% and long COVID-19 by 21% (1). The underestimation of predictions can be plausibly explained by the arrival of the Omicron variants and the changes in public health policies in the UK (2–4).

Dynamic Causal Modeling (DCM) besides predicting health outcomes can also estimate changing characteristics of the epidemic, such as the properties of viral transmission, immunity induced by vaccine or infection, and the propensity to leave home and increase the risk of catching the infection. Do these predictions corroborate the assumption that the underestimations were due to new variants and the changes in the use of non-pharmaceutical interventions.

This paper is a sequel to the previous paper predicting 12-months to October 2022. It sets out to assess the underlying properties of the epidemic during that period. It also seeks to predict what will happen in the 12 months to October 2023 assuming the current properties of the epidemic remain as they are in October 2022.

## Methods

### Dynamic Causal Models

The Dynamic Causal Model (DCM) used in this research has been continually updated with data as the epidemic has unfolded. It is designed to allow modification of model parameters, such as transmissibility of the virus, changes in social distancing, and vaccine coverage—to accommodate changes in population dynamics and virus behavior. A recent model (1st October 2022) was used to explore the effect of increased ease of transmission of the Omicron variants and the likely seasonal effect of the coming winter. The potential benefit of a successful Find, Test, Trace, Isolate and Support scheme was also incorporated into the model.

#### General features of DCMs

Standard SEIR models tend to offer quantitative epidemiological forecasts that rest upon fitting curves to the recent trajectory of various epidemiological data; e.g., (5, 6). Some researchers have augmented SEIR models by incorporating population-based behavior data such as Google mobility to measure adherence to non-pharmaceutical interventions. These changes of behavior are modeled to affect transmission characteristics through changing contact rates (7). Other researchers have used state space modeling like ours (8–10), but not with the added features of the Dynamic Causal Model which include a form of agent-based behavioral modeling. In other words, the conventional SEIR model is absorbed into a larger state-space model that accounts for changes in behavior and testing. As such, Dynamic Causal Modeling can predict *mitigated* outcomes and quantify the uncertainty associated with those outcomes. It considers what is most likely to happen, based upon a generative model that best explains all the data available. This mandates a model of socio-behavioral responses that mitigate viral transmission, such as social distancing, lockdown, testing and tracing, *etc*.

#### Specific features of this DCM model

The model is fully described as a supplement to this article. There is a weekly dashboard which provides up-to-date estimates and projections (11). The software is freely available and can be run using datasets from other countries (12–16).

How does this DCM model deal with the effect of sociobehavioural responses on contact rates and ensuing viral spread? In the model contact rates depend upon location (i.e., being at home vs. being at work), where movement between locations depends upon prevalence (i.e., the probability of leaving home for work reduces monotonically with prevalence). This construction means that we assume people infer the prevalence of infection at any given time and adjust their behavior accordingly. We do not model the mechanisms of this inference but simply parameterize the sensitivity of behavioral responses to prevalence (i.e., the probability of being in an infected or infectious state). This assumes that people assimilate the evidence for prevalence (from media reports, dashboards, incidence of infection among family and friends, et cetera) and—averaged over the entire population—converge on an unbiased estimate (17). Please see Supplementary material for the functional form of this prevalence-dependent effect. This functional form emerged *via* Bayesian model comparison, in which we compared models that conditioned behavioral responses on combinations of latent states (e.g., prevalence, incidence, hospitalization, fatality rates, et cetera) and plausible non-linear functions.

The model includes all the standard SEIR (susceptible, exposed, infected, removed) features of the commonly used models of infectious disease but in addition incorporates the interactions between the different variables. For example, people are more likely to stay at home if prevalence is high or if they have not been immunized. These dependencies are estimated and only retained if they improve the ability of the model to account for the data. Having optimized the model and model parameters, one can then proceed with scenario modeling to evaluate the effect of interventions such as the influence of an enhanced Find, Test, Trace, Isolate and Support system on the epidemic.

Standard SEIR models depend on the choice of parameters, some of which are unknown empirically and must be guessed. Dynamic Causal Modeling is, by comparison, relatively assumption free. However, one must specify prior ranges for parameters (just like for SEIR models) but the DCM adjusts the parameters to fit the data in the most efficient and parsimonious way possible. Not only does the model provide estimates and projections of variables such as the death rate, the effective reproductive number, incidence, and prevalence but it also estimates transmissibility, susceptibility, latent resistance, herd immunity, expected social distancing behavior, and vaccine effectiveness. For example, the model includes three parameters in respect of population immunity; one is the loss in antibody-related immunity in days induced by COVID-19 infection or vaccine, another is the loss of T-cell immunity in days induced by COVID-19 infection and the third is the proportion of the population that is naturally immune to the infection (Supplementary Figure S2). While the first two change with time (as vaccines and the virus change) the natural resistance is assumed to remain constant.

### Data sources and assumptions

The latest data from the UK Health Security Agency (UKHSA) (18) and the COVID-19 Infection Survey of the Office of National Statistics (19) were used, together with the Google Mobility Report (20). The Oxford stringency index was used as a measure of the changing use of national non-pharmaceutical interventions (3). IHME provided an up to date estimate of national incidence (21). For the predictions to October 2023, it is assumed that mitigation efforts in schools and workplaces will not take place, that lockdown will not be re-imposed, and that no new more virulent variant will arrive.

## Findings

### Finding plausible reasons for underestimating the wave of infections between October 2021 and October 2022

#### The size of the underestimation

The projected total number of new COVID-19 cases was underestimated by 43% (1). In-person testing at test sites and free lateral flow device tests became no longer available on 1st April 2022. While it was predicted that 26% of cases would be confirmed by a PCR test, in the event only 21% were so confirmed. The total number of tests carried out was only overestimated by 24% which can be explained by the discontinuation of free LFD tests. Deaths were underestimated by 20%. Hospital admissions were underestimated by 31%. Long COVID-19 was underestimated by 21%.

#### Changing characteristics of the variants in circulation

**Figure 1:**
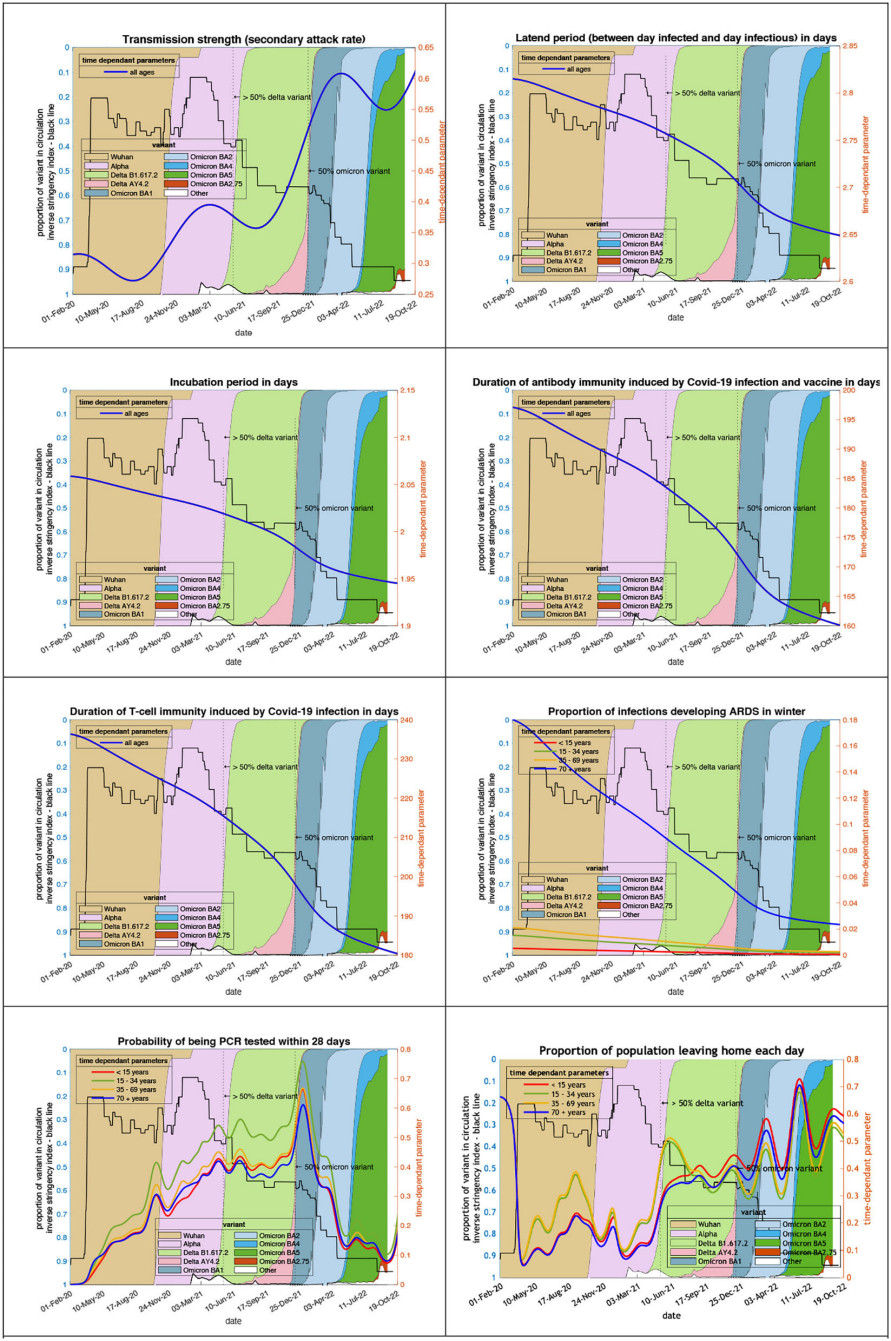
Changing estimates of transmission and immune profiles in relation to the emergence of new variants and changes in response to public health policies: UK February 2020 to October 2022. Note: Transmission strength is measured as the secondary attack rate with prior value of 0.2 (i.e., an infected person infects 1 in 5 contacts), which rises with the newer variants to 0.5 (i.e., one infection infects 1 in 2 contacts); antibody immunity which falls with newer variants is measured in days of waning.

The transmission strength and other epidemiological variables in Figure 1 are estimates that best explain the surveillance and hospital data. This figure shows the systematic changes in key epidemiological parameters, such as transmission strength and incubation period. Some show slow systematic trends over time, while others—such as the propensity to “leave home” that summarizes sociobehavioural responses— fluctuate markedly with the emergence of new variants. Crucially, the model did not “know about” the variants (shaded regions) and yet their effects are clearly manifest in terms of sociobehavioural responses to fluctuations in transmissibility and prevalence. Transmission has become more likely as measured by the secondary attack rate which has increased from 0.2 in March 2020 (i.e., an infected person infects 1 in 5 contacts) to 0.4 on 1st October 2021 to 0.5 (i.e., one infection infects 1 in 2 contacts) in October 2022. The speed of infection has also increased with (i) the latent period shortening (between the day infected and the day infectious) from 2.8 at the start of the epidemic to 2.7 by October 2021 to 2.6 in October 2022, and (ii) the incubation period shortening from 2.05 in March 2020 to 2.0 in October 2021 to 1.95 in October 2022.

Immunity has waned throughout the epidemic. Antibody immunity induced by COVID-19 infection and vaccines has waned from 197 in March 2020 to 177 in October 2021 to 160 days in October 2022. T-cell immunity has also waned from 236 in March 2020 to 205 in October 2021 to 180 days in October 2022.

The pathogenicity of the variants has also changed since the Wuhan variant. The proportion of infections developing acute respiratory distress syndrome has fallen from 18% in April 2020 to 6% in October 2021 to 2% in October 2022.

#### Changing public health responses

Testing for COVID-19 has become uncommon in the UK. The probability of 35- to 69-year-olds being PCR positive within 28 days of COVID-19 infection has dropped from 0.033 at the peak in December 2021 to 0.003 in October 2022. The public response to the dropping of public health interventions despite the wave of infections has been an increase in the population leaving home each day—a measure of placing oneself in a high-risk environment—from 4% during the first lockdown in April 2020 to 33% in October 2021 to 58% in October 2022. The trend is a mirror image of the stringency index.

Having quantified the predictive validity of long-term forecasts between October 2021 to October 2022 using known outcomes, we now turn to predictions over the forthcoming year from October 2022 to October 2023 and take the opportunity to consider predictions under different public health measures.

### Model predictions to October 2023

The posterior predictions produced by the model assume that certain parameters—such as the properties of the latest variants, the vaccine coverage and the use of non-pharmaceutical interventions—remain as before. They provide a counterfactual to compare with the effect of future variants and non-pharmaceutical interventions.

**Figure 2:**
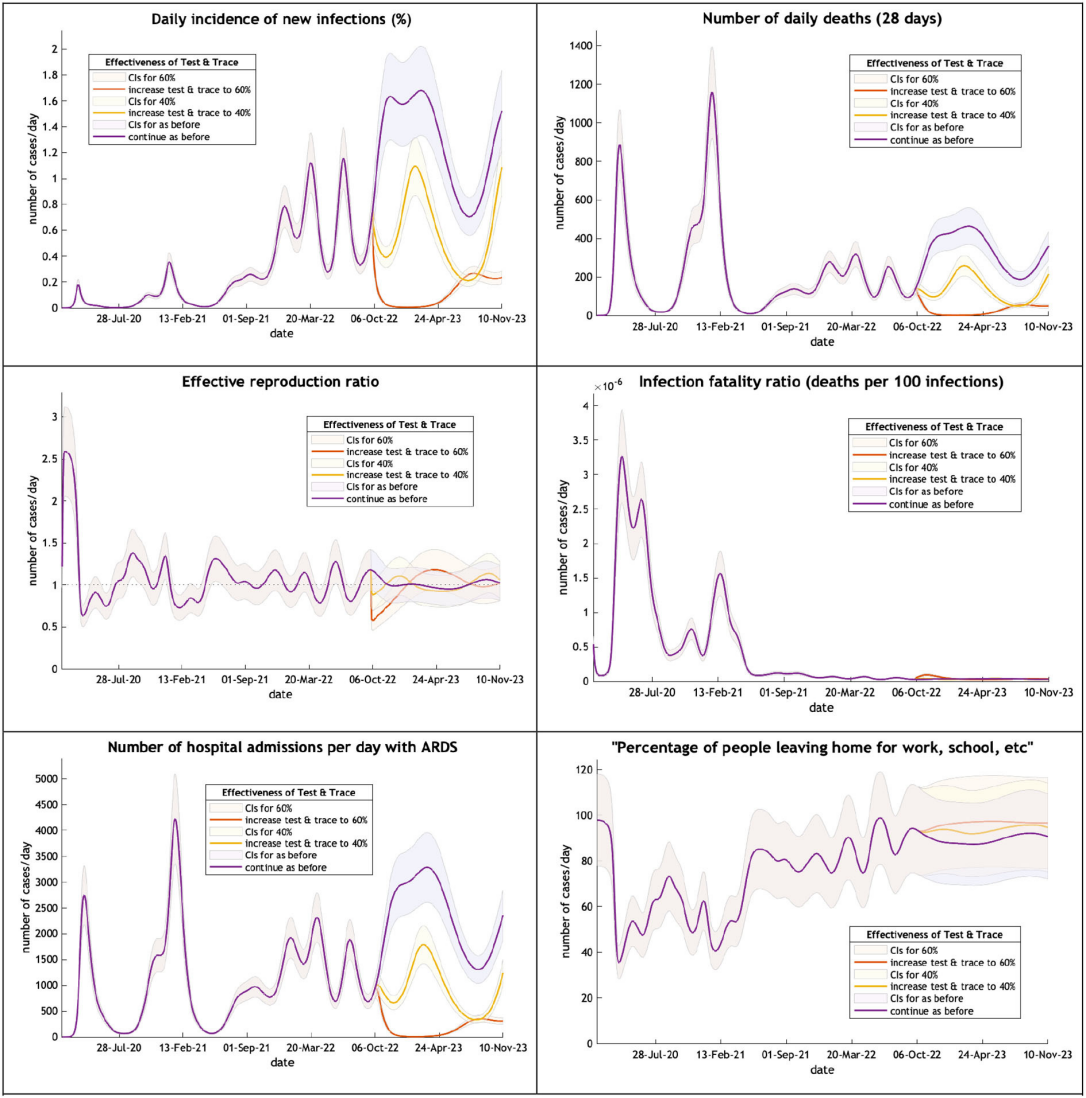
The UK epidemic curve from February 2020 to October 2023—incidence, deaths, the effective reproductive ratio, the infection fatality ratio, hospital admissions and mobility. Note: Each figure offers three projections: purple if the test & trace system remains as now (24% effective), yellow if the test & trace improves effectiveness to 40%, orange to 60% on 1st October 2022. CIs, 90% credible intervals.

The ensuing predictions illustrate the depth and persistence of the future epidemic in the UK in terms of morbidity and mortality, transmission characteristics, testing capacity, hospital utilization, and disruption due to acute and chronic symptoms over the next 12 months if the government continues to withhold public health infection control measures (Figure 2).

A large wave (purple line) of COVID-19 is expected over the winter months peaking on 24th February next year with new infections affecting 1.9% of the population. This will produce an increase in hospital admissions but because of a low infection fatality rate little increase in critical care beds and fewer deaths than might have been expected. Mobility will remain above 90% of the pre-epidemic level. A modest improvement in the Find, Test, Trace, Isolate and Support (yellow and orange lines) would dramatically reduce new cases and their sequelae.

### Long term consequences

The model can calculate the cumulative effect of the epidemic on case numbers, deaths, tests, hospital admissions and long COVID (Table 1).

**Table 1:** The cumulative effect of COVID-19 to October 2023 projected by the DCM on 1^st^ October 2022 for UK.

**Cumulative totals from 1st February 2020 to**	**1st October 2022**		**07-Oct-22**	**1st Oct 2023**
**Scenario assuming FTTIS is 25% effective**	**Actual**	**Data source**	**DCM estimates**	**DCM projection**
Estimated incidence	105,678,303	IHME	177,323,347	485,603,813
Confirmed cases by PCR	22,241,311	UK COVID-19 dashboard	23,178,802	53,409,837
Deaths within 28 days of a positive PCR test	177,977	UK COVID-19 dashboard	209,474	330,957
Tests (both PCR and LFD)	514,605,757	UK COVID-19 dashboard	549,139,089	821,181,901
Hospital admissions	993,657	UK COVID-19 dashboard	1,019,852	1,867,580
Acute-post-COVID-19 Syndrome ≥12 weeks	1,725,968	ONS Infection survey	1,725,968	4,726,602

A 3 fold increase in the number of new cases in the next 12 months is predicted. This has the effect of increasing COVID-19 deaths by 120,000 in the coming year which is half the number of deaths which have been caused by COVID-19 so far. The new wave of COVID-19 is likely to add 800,000 hospital admissions and for the number of people suffering acute-post-COVID-19 syndrome to reach 4.7 million.

## Interpretation

The transmission strength and other epidemiological variables in Figure 1 are estimates that best explain surveillance and hospital data. The alignment between changes in these estimates and the emergence of new variants lends them a predictive and construct validity. In this sense, the emergence of new variants could plausibly be predicted from fluctuations in estimated transmission strength and subsequent socio-behavioral responses (i.e., the probability of leaving home in the last panel). The trend of these parameters chosen by the model to best fit the data helps to explain the underestimations of the projections produced by the model in October 2021. The results are a reminder that the model can only predict what is likely to happen, looking forward 12 months, if key features of the epidemic remain unchanged or change in a systematic and predictable way.

The Dynamic Causal Model can produce a counter-factual to compare with alternative policy options. The ability to predict what will happen if public health responses remain the same is particularly useful if the predictions seem to be reasonable as found in last year's projections. They allow one to get a feel for the scale of the ensuing epidemic. In this case a very large wave of new infections is predicted for the period to October 2023 and while severe illness and death are not so common with Omicron variants as Delta, the numbers add up to a substantial toll. The scale of the potential long COVID epidemic is difficult to grasp. Together with illness due to the acute infection the new wave is likely to have a serious effect on the economy in terms of sickness absence and long-term debility.

A competent Find, Test, Trace, Isolate and Support would reduce considerably the size of the projected wave and its sequelae. A vaccine alone policy will be insufficient. Additional mitigation would relieve the economy. Lockdowns and additional critical care beds are not required.

The precautionary principle, a major feature of many respected public health policies, is provoked by these findings. If the size of the predicted wave was small and insignificant there would be no need to initiate and prepare for more active interventions. But the risks of doing nothing, with hospitals running at full capacity, and having no proper Find, Test, Trace, Isolate and Support system in place and ready to be expanded rapidly are substantial. A new variant with more pathogenic features is a real possibility. An exhausted NHS exacerbated by sickness absence is likely.

The main limitation of the study is that few epidemiologists have used Dynamic Causal Models and sought to replicate our methods and results. The model and methodology are freely available and ready to be verified. A technical limitation of this application of Dynamic Causal Modeling is that the estimates are predicated on a single model. This means that the uncertainty—inherent in long-term projections—only reflects uncertainties about the parameters of the model. However, there would be additional uncertainty about the (structure of the) model *per se*, had we considered several models. An exhaustive search of all plausible models is beyond the reach of our (academic) resources. However, in principle, the very structure and assumptions of the model could be explored using Bayesian model comparison, should this approach to epidemiological modeling be adopted more widely. At this early stage in their use in understanding infectious epidemics Dynamic Causal Models can be considered as alternative and additional to standard and well-understood SEIR models.

In conclusion, the 12-month projection in 2021 underestimated the scale of the subsequent wave of infections. The 2023 projection could well-underestimate the size of the next wave of COVID-19 infection which we predict will be extensive. Far from thinking that the COVID-19 epidemic is over, the cautious approach might be to apply mitigation measures now, plan for a new and more virulent variant and prepare the population for what might well be a difficult situation in 2023.

## Data availability statement

The datasets presented in this study can be found in online repositories. The names of the repository/repositories and accession number(s) can be found below: https://figshare.com/articles/Dynamic_Causal_Modelling_of_COVID-19/1217; https://www.dropbox.com/sh/jyr6dzb77wcyox0/AAD80PzpX0iaXDmte95HKztja?dl=0.

## Ethics statement

Ethical review and approval was not required for the study on human participants in accordance with the local legislation and institutional requirements. Written informed consent for participation was not required for this study in accordance with the national legislation and the institutional requirements.

## Author contributions

KF designed and used the Dynamic Causal Model to research features of the COVID-19 pandemic for over 2 years. CB used the latest version of the model to explore the predicted epidemiology of the epidemic in the UK, wrote the first draft, and was the submitting author. Both authors contributed to the article and approved the submitted version.
